# Increased accessibility of computer-based testing for residency application to a hospital in Brazil with item characteristics comparable to paper-based testing: a psychometric study

**DOI:** 10.3352/jeehp.2024.21.32

**Published:** 2024-11-11

**Authors:** Marcos Carvalho Borges, Luciane Loures Santos, Paulo Henrique Manso, Elaine Christine Dantas Moisés, Pedro Soler Coltro, Priscilla Costa Fonseca, Paulo Roberto Alves Gentil, Rodrigo de Carvalho Santana, Lucas Faria Rodrigues, Benedito Carlos Maciel, Hilton Marcos Alves Ricz

**Affiliations:** 1 Ribeirão Preto Medical School, University of São Paulo, Ribeirão Preto, Brazil; 2 eduCat, Belo Horizonte, Brazil; 3 Ribeirao Preto Clinical Hospital, Ribeirão Preto, Brazil; Hallym University, Korea

**Keywords:** Computers, COVID-19, Educational measurement, Feasibility studies, Internship and residency, Brazil

## Abstract

**Purpose:**

With the coronavirus disease 2019 pandemic, online high-stakes exams have become a viable alternative. This study evaluated the feasibility of computer-based testing (CBT) for medical residency applications in Brazil and its impacts on item quality and applicants’ access compared to paper-based testing.

**Methods:**

In 2020, an online CBT was conducted in a Ribeirao Preto Clinical Hospital in Brazil. In total, 120 multiple-choice question items were constructed. Two years later, the exam was performed as paper-based testing. Item construction processes were similar for both exams. Difficulty and discrimination indexes, point-biserial coefficient, difficulty, discrimination, guessing parameters, and Cronbach’s α coefficient were measured based on the item response and classical test theories. Internet stability for applicants was monitored.

**Results:**

In 2020, 4,846 individuals (57.1% female, mean age of 26.64±3.37 years) applied to the residency program, versus 2,196 individuals (55.2% female, mean age of 26.47±3.20 years) in 2022. For CBT, there was an increase of 2,650 applicants (120.7%), albeit with significant differences in demographic characteristics. There was a significant increase in applicants from more distant and lower-income Brazilian regions, such as the North (5.6% vs. 2.7%) and Northeast (16.9% vs. 9.0%). No significant differences were found in difficulty and discrimination indexes, point-biserial coefficients, and Cronbach’s α coefficients between the 2 exams.

**Conclusion:**

Online CBT with multiple-choice questions was a viable format for a residency application exam, improving accessibility without compromising exam integrity and quality.

## Graphical abstract

[Fig f4-jeehp-21-32]

## Introduction

### Background

High-stakes exams are common in health professions education and intend to generate a minimum or adequate score or concept related to decision-making, such as admissions, approvals, licensing, and maintenance of certification [1].

The coronavirus disease 2019 (COVID-19) pandemic brought several challenges for in-person evaluations, especially for large-scale exams that required traveling and could involve large groups of people gathering in confined spaces. This led to the need for structural rearrangements to provide sufficient physical distance and even the cancellation of exams. Technological improvements have made online high-stakes computer-based testing (CBT) a viable alternative. Compared to traditional paper-based testing, internet-based CBT with multiple-choice question (MCQ) items has some advantages, such as efficiency, immediate scoring and feedback, ease of item analysis, and similar exam scores [2,3]. Conversely, online proctoring has raised concerns about security, privacy, ethics, and educational experience [4,5]. It is a challenge to balance the use of proctored exams with pedagogical principles, and there is still a lack of studies assessing the impact of internet-based CBT with MCQ items, especially for medical residency application exams in hospitals in Brazil.

### Objectives

This study aimed to assess the feasibility of a high-stakes online CBT with MCQ items in a medical residency application to Ribeirao Preto Clinical Hospital during the COVID-19 pandemic and to evaluate its potential impacts on accessibility for exam candidates and item quality.

## Methods

### Ethics statement

The study did not need to be approved by the institutional review board because it was based on examination results without any individual identification.

### Study design

This psychometric study was conducted in the Clinical Hospital of Ribeirao Preto Medical School in Brazil. It was described according to the STROBE (Strengthening the Reporting of Observational Studies in Epidemiology) statement, available at https://www.strobe-statement.org.

### Setting

Brazil is a continental country divided geographically into 5 distinct regions: North, Northeast, Center-West, Southeast, and South. The Brazilian regions have significant socioeconomic inequalities and income disparities, with the North and Northeast being the most deprived regions and the Southeast the richest and most populated [6].

Residency programs in Brazil can be divided into direct-access programs (internal medicine, surgery, pediatrics, obstetrics & gynecology, and public health) and programs with prerequisites. Admission to the programs, regulated by the National Committee of Medical Residency, occurs via public competition and comprises a maximum of 3 phases: cognitive assessment (mandatory), skill assessment (optional), or curriculum review (optional) [7]. The Clinical Hospital of Ribeirao Preto Medical School (HCFMRP), University of Sao Paulo, is located in the state of Sao Paulo, in the Southeast region, and is one of Brazil’s most significant institutions in terms of the numbers of medical residency positions and programs. As the direct-access programs are very competitive, applicants need to take 3 phases: an exam with MCQ items, an objective structured clinical examination, and a curriculum review.

Before the COVID-19 pandemic, the cognitive assessment was performed as a paper-based test (PBT). During the pandemic, especially when virus transmission was uncontrolled, some cities declared lockdowns and adopted strict social distancing, potentially leading to the cancellation of public competitions. In 2020, we opted to perform the cognitive assessment as a web-based exam for all programs.

### Proctoring of the web-based exam

Several aspects of the online platform and assessment design were considered to combat cheating and maintain security, academic integrity, and pedagogical principles. All candidates needed to take the exam on a personal computer equipped with a microphone and a webcam in an isolated room. They were required to install a lockdown browser that accessed the microphone and the webcam and prevented any access to other websites.

Items were displayed randomly among candidates, one at a time, and backtracking was not allowed. Candidates had a predetermined time (2.5 minutes) to answer each item. Remote, live, and artificial intelligence proctoring occurred throughout the entire exam.

### Variables

Item’s psychometric characteristics of items based on the item response theory (IRT) and classical testing theory (CTT) were variables.

### Data sources and measurement

#### Computer-based testing with MCQ items

In 2020, the internet-based CBT with MCQ items was adopted as the cognitive assessment for all medical residency applicants at HCFMRP. For the direct-access program, 120-MCQ items on the main 5 areas (internal medicine, surgery, pediatrics, obstetrics & gynecology, and public health) were constructed similarly to the previous years, with specialists from all 5 areas. The exams were administered through internet-based CBT developed by eduCat (https://educat.com.br/). Two weeks before, all applicants were required to log into the online CBT platform and undergo practical training about all functionalities and rules. The internet connection quality was also measured. Response data are available in Dataset 1. Dataset 2 contains the correct answers for the 120 items.

#### Paper-based testing with MCQ items

Two years later (2022), the cognitive assessment for all medical residency applicants returned to a PBT. The item construction process was similar to the CBT, except that the exam had 100-MCQ items in the 5 main areas (internal medicine, surgery, pediatrics, obstetrics & gynecology, and public health) instead of the 120 items in the CBT. Response data are available in Dataset 3, and Dataset 4 contains the correct answers for the 100 items.

#### Psychometric analysis

The difficulty index, expressed as the percentage of applicants with the correct answers to the total number of applicants ranges from 0% to 100% or 0.00 to 1.00. It was categorized as very easy (0.85 to 1.00), easy (0.65 to 0.84), moderate (0.35 to 0.64), difficult (0.15 to 0.34), or very difficult (0.00 to 0.14). The discrimination index, which indicates the ability of an item to differentiate high-performing versus low-performing applicants, was calculated based on the difference between the 27% upper group and the 27% lower group. It was defined as negative, poor, moderate, good, or excellent using the following cutoffs: -1.00 to 0.09, 0.10 to 0.19, 0.20 to 0.29, 0.30 to 0.39, and 0.40 to 1.00, respectively. The point-biserial coefficient ranges from -1 to +1 and indicates the association between an applicant’s performance on a specific item and the applicant’s overall performance. It was categorized as negative (-1.00 to -0.99), inadequate (0.00 to 0.09), very low (0.10 to 0.19), low (0.20 to 0.29), good (0.30 to 0.39), or very good (0.40 to 1.00). Difficulty and discrimination indexes and point-biserial coefficients were calculated based on the CTT. We also computed difficulty and discrimination indexes and guessing based on the IRT using the 3-parametric likelihood (PL) model, with parameters a (discrimination), b (difficulty), and c (guessing). Reliability was calculated using the Cronbach’s α coefficient.

### Bias

There was no bias in selecting participants. All participants’ responses to items were analyzed.

### Statistical methods

Descriptive statistics were used to summarize the number of programs, applicants’ demographics, dishonest behavior, and Cronbach’s α reliability index. Data were reported as percentages and means±standard deviations. The region of applicants’ medical school, discrimination index, difficulty index, point-biserial coefficient according to the CTT and difficulty parameter, discrimination parameter, and guessing parameter according to the IRT were calculated. We used R (https://www.r-project.org/) for CTT, IRT, and Cronbach’s α reliability index calculations (R packages included mirt, mirtCAT, and psych). The R code used for this study is provided in Supplements 1–3. GraphPad Prism software ver. 10.0 (GraphPad- Prism Software Inc.) was used for all statistical analyses.

## Results

### Demographic characteristics

In 2020, 4,846 individuals (57.1% female, mean age of 26.64±3.40 years) applied to the direct-access program, and 3,223 (66.5%) took the internet-based CBT. In 2022, 2,196 individuals (55.2% female, mean age of 26.47±3.20 years) applied to the direct-access program, and 1,994 (90.8%) took the PBT. There were no significant differences in demographic characteristics between applicants of the 2 tests (P>0.05). Compared to the PBT, the CBT had an increase of 2,650 applicants (120.7%), but 66.5% completed the CBT versus 90.8% completed the PBT.

Applicants for the CBT were from 272 different medical schools distributed among all 26 Brazilian states, Federal Districts, and foreign institutions. In contrast, applicants for the PBT were from 235 medical schools distributed in 25 Brazilian states, Federal Districts, and foreign institutions (Table 1).

### Item characteristics

**Fig. 1.**
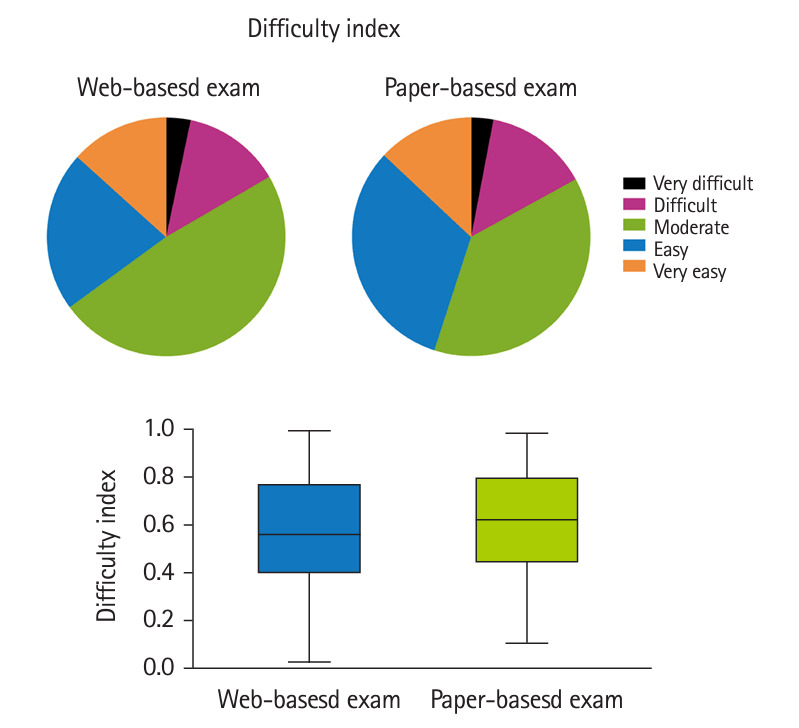
Distribution of items according to the difficulty index in the web-based and paper-based exams.

**Fig. 2.**
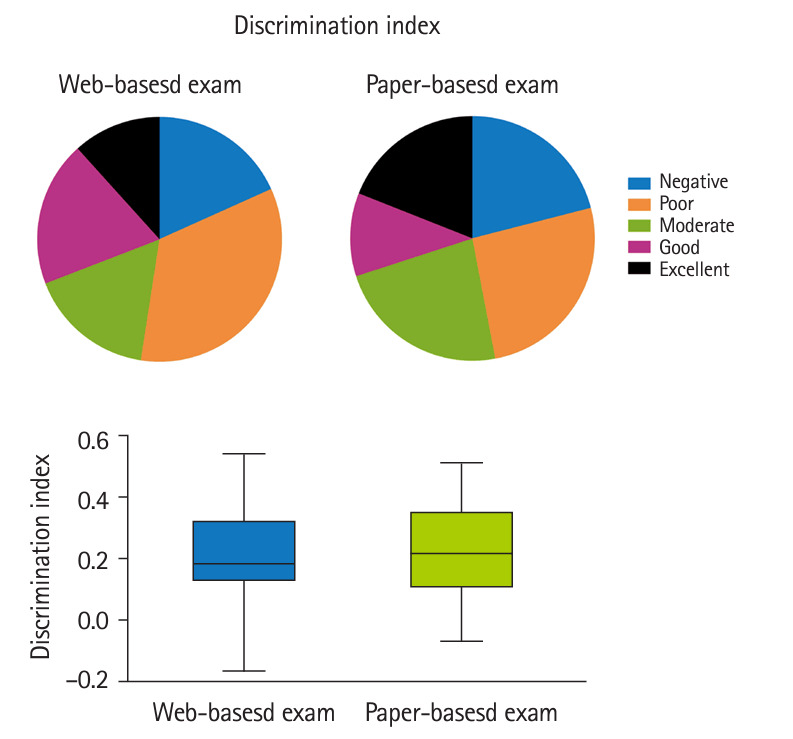
Distribution of items according to the discrimination index in the web-based and paper-based exams.

**Fig. 3.**
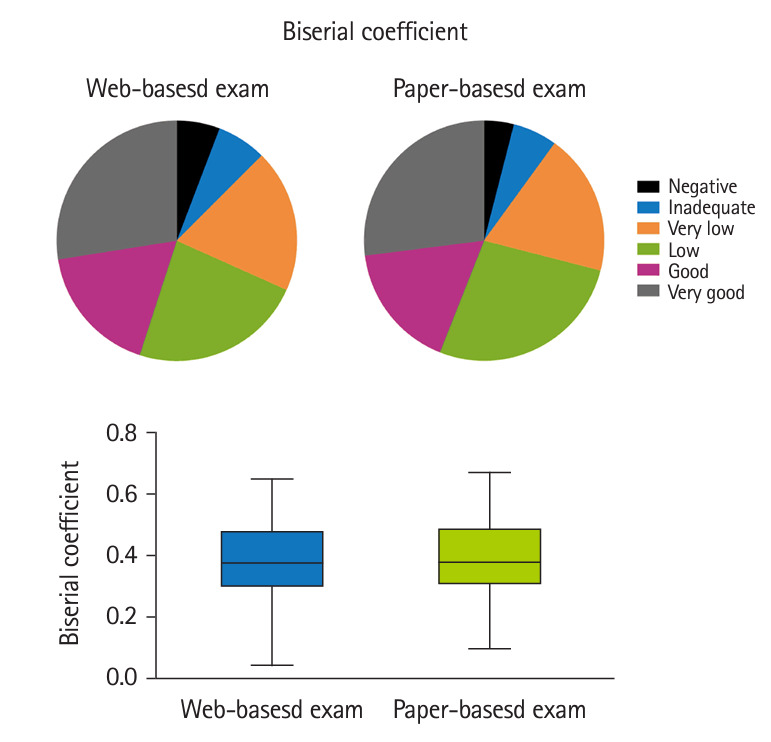
Distribution of items according to the point-biserial coefficient index in the web-based and paper-based exams.

The CTT analysis showed no significant differences in the difficulty and discrimination indexes and point-biserial coefficient between the web-based and in-person exams (Figs. 1–3, Table 2). In both tests, the majority of items had intermediate and easy difficulty levels; the very difficult, difficult, and very easy items also had a similar percentage in both exams (Fig. 1). The discrimination index varied from 18.3% and 21.0% of items with negative discrimination to 16.7% and 23.0% with moderate discrimination in the web-based and in-person exams, respectively (Fig. 2). There was a slightly but non-significantly higher percentage of items with excellent discrimination in the PBT than in the CBT (19.0% versus 11.7%). Both exams had a similar distribution of point-biserial coefficient (Fig. 3). The results of item analysis based on the IRT are presented in Table 2. Cronbach’s α, as an indicator of internal consistency reliability, was 0.86 and 0.85 for the online and in-person exams, respectively.

### Stability and consistency of Internet for CBT

The web-based exam occurred without interruptions. Concomitant real-time psychometric analyses helped in decision-making. Only 3 applicants (0.09%) had internet instability, and 2 (0.06%) were excluded for cheating.

## Discussion

### Key results

Amidst the COVID-19 pandemic, a residency application exam for a hospital in Brazil was successfully performed. Of note, with the CBT, the number of applicants, mostly from distant and poor regions, significantly increased. These results underscore that online CBT can be a tool to facilitate access and decrease inequity in high-stakes exams.

### Interpretation/comparison with previous studies

Several concerns about online CBT have been raised, such as exam misconduct, security, ethics, internet technical problems, educational experience, and changes in exam quality [5]. Previous studies have pointed out that unequal access to technology and digital inequality could decrease learning proficiency and the fairness of online exams [5]. In our study, 3,223 applicants from all Brazilian states could take the CBT with MCQ items, and only 3 applicants had internet instability, showing that technical difficulties were not a problem even in a large country. In our online CBT, we had more applicants, and they were from more distant and poorer regions, facilitating applicants’ access and, consequently, exam fairness. With online CBT, applicants do not need to travel to test centers, potentially reducing overall costs.

Other studies have shown that students who experience online exams consider them easier to take and more economical [8]. During the COVID-19 pandemic, a comprehensive high-stakes online exam for final-year dental students was implemented successfully with good satisfaction by students and faculty members but presented minor technological issues [9]. In another study, 70.3% of students showed several concerns and negative perceptions about remote e-exams before the exam; however, after participating in online exams, their perceptions changed to more positive ones [10]. Important issues students raise are the prevention of backtracking, unjustified invalidation of their exams, background noise, webcam and internet problems, and privacy [11].

Most studies that looked for the impact of online CBT on students’ results demonstrated that performance did not differ significantly between PBT and online CBT [12]. Jaap et al. [12] showed that a remote online CBT with MCQ items for summative assessment was effective and acceptable for medical students, with few students experiencing problems without detriment to candidate performance. In a study that used remote proctoring in a proficiency test for admission to the Advanced Master of General Practice, exam results were similar to the on-site proctored exam. Still, remote proctoring was associated with mixed feelings [13].

Not all studies assessed the impact of proctoring and test delivery mode (CBT versus PBT) on item quality. In our CBT, item quality, demonstrated by the difficulty and discrimination indices, point-biserial coefficient, and Cronbach’s α coefficient, did not significantly differ from that of PBT.

### Limitations

The number of items and content were different in the 2 tests. In Brazil, all test items must be provided to applicants immediately after the exam due to legal requirements. Since applicants typically solve the previous items to prepare for the next one, we were unable to use common items to equate the 2 tests for the IRT analysis. The equating process with common item content should be conducted for IRT analysis. It is impossible to compare the item characteristics from 2 tests by item analyses according to IRT. This is the reason why only the comparison of item characteristics by CCT was provided.

### Conclusion

Internet-based CBT was a viable format for large applicants to a hospital’s residency program, enhancing accessibility without compromising exam integrity and quality.

## Figures and Tables

**Figure f4-jeehp-21-32:**
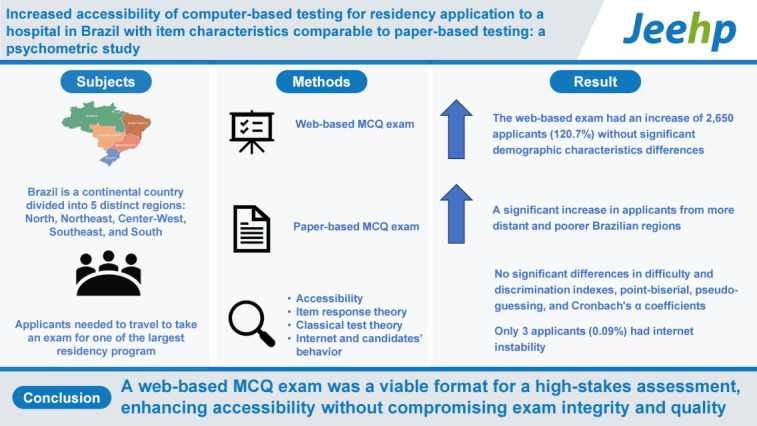


**Table 1.** Number of direct-access programs, applicants, and applicants’ medical schools in web-based and paper-based exams

Variable	2020	2022
	Web-based exam	Paper-based exam
No. of direct-access programs	21	22
No. of new positions each year	172	185
No. of applicants	4,446	2,196
No. of applicants who completed the exam	3,223	1,994
Applicant/position ratio	25.8	11.9
No. of medical schools	273	235
Regions of medical schools^a)^		
South	462 (9.5)	169 (7.7)
Southeast	2,782 (57.4)	1,553 (70.7)
Center-West	476 (9.8)	203 (9.2)
Northeast	821 (16.9)	198 (9.0)
North	273 (5.6)	59 (2.7)
Foreign	32 (0.7)	14 (0.6)

Values are presented as number or number (%).

a) P<0.05.

**Table 2.** Difficulty and discrimination indexes, point-biserial coefficient, guessing, and Cronbach’s α coefficient in a web-based and paper-based exam according to the item response and classical test theories

Variable	2020	2022
	Web-based exam	Paper-based exam
Classical test theory		
Difficulty index^a)^	0.57±0.22	0.60±0.22
Discrimination index^a)^	0.22±0.14	0.23±0.14
Point-biserial coefficient^a)^	0.29±0.17	0.29±0.16
Item response theory		
Difficulty parameter	0.03±1.60	0.29±1.54
Discrimination parameter	1.04±0.38	1.11±0.41
Guessing parameter	0.20±0.08	0.21±0.08
Internal consistency reliability		
Cronbach’s α	0.86	0.85

Values are presented as mean±standard deviation unless otherwise stated.

a) Not significant (P>0.05).

## Footnotes

**Data availability**
Data are available from Harvard Dataverse: https://doi.org/10.7910/DVN/YJP1ZM Dataset 1. Raw responses of 3,223 applicants to 120 multiple-choice items of computer-based testing for residency in a hospital in Brazil.

Dataset 2. Correct options for 120 items of the computer-based testing.

Dataset 3. Raw responses of 1,994 applicants to 100 multiple choice items of paper-based testing for residency in a hospital in Brazil.

Dataset 4. Correct options or 100 items for paper-based testing.

## Supplementary materials

Supplementary files are available from Harvard Dataverse: https://doi.org/10.7910/DVN/YJP1ZM

Supplement 1. R code for the unidimensionality test.

Supplement 2. R code for the item analyses based on the classical test theory.

Supplement 3. R code for the item analyses based on the item response theory.

Supplement 4. Audio recording of the abstract.
